# Distal radioulnar joint stabilization with open foveal reinsertion versus tendon graft reconstruction: an experimental study using radiostereometry

**DOI:** 10.1186/s40634-021-00329-y

**Published:** 2021-02-04

**Authors:** Janni Kjærgaard Thillemann, Sepp De Raedt, Torben Bæk Hansen, Bo Munk, Maiken Stilling

**Affiliations:** 1 University Clinic for Hand, Hip and Knee Surgery, Hospital Unit West, Lægaardvej 12, 7500 Holstebro, Denmark; 2 Department of Clinical Medicine, Aarhus University, Palle Juul-Jensens Boulevard 165, 8200 Aarhus N, Denmark; 3 NRT X-RAY A/S, Birkegårdsvej 16, 8361 Hasselager, Denmark; 4 Department of Orthopaedic Surgery, Aarhus University, Palle Juul-Jensens Boulevard 165, J-501, 8200 Aarhus N, Denmark

**Keywords:** Distal radioulnar joint, Instability, Radiostereometry, Reconstruction, Surgery, Triangular fibrocartilage complex

## Abstract

**Purpose:**

Symptomatic instability of the distal radioulnar joint (DRUJ) caused by lesion of the Triangular Fibrocartilage Complex (TFCC) can be treated with a number of surgical techniques. Clinical examination of DRUJ translation is subjective and limited by inter-observer variability.

The aim of this study was to compare the stabilizing effect on DRUJ translation with two different surgical methods using the Piano-key test and a new precise low-dose, non-invasive radiostereometric imaging method (AutoRSA).

**Methods:**

In a randomized experimental study we evaluated the DRUJ translation in ten human cadaver arms (8 males, mean age 78 years) after cutting the proximal and distal TFCC insertions, and after open surgical TFCC reinsertion (*n* = 5) or TFCC reconstruction using a palmaris longus tendon graft ad modum Adams (*n* = 5).

The cadaver arms were mounted in a custom-made fixture for a standardized Piano-key test. Radiostereometric images were recorded and AutoRSA software was used for image analyses. Standardised anatomical axes and coordinate systems of the forearm computer tomography bone models were applied to estimate DRUJ translation after TFCC lesions and after surgical repair.

**Results:**

The DRUJ translation after cutting the proximal and distal TFCC insertions was 2.48 mm (95% CI 1.61; 3.36). Foveal TFCC reinsertion reduced DRUJ translation by 1.78 mm (95% CI 0.82; 2.74, *p* = 0.007), while TFCC reconstruction reduced DRUJ translation by 1.01 mm (95% CI -1.58; 3.60, *p* = 0.17).

**Conclusion:**

In conclusion, foveal TFCC reinsertion significantly decreased DRUJ translation while the stabilizing effect of Adams TFCC reconstruction was heterogeneous. This supports the clinical recommendation of TFCC reinsertion in patients suffering from symptomatic DRUJ instability due to acute fovea TFCC lesions.

## Background

Symptomatic instability of the distal radioulnar joint (DRUJ) can result from lesion of the DRUJ stabilizing Triangular Fibrocartilage Complex (TFCC). Nakamura et al. described that the ulnar-sided TFCC insertion consist of both a distal component (dc) at the ulnar styloid and a proximal component (pc) at the ulnar fovea [22]. The pc-TFCC lesion is associated with a higher degree of DRUJ instability than the dc-TFCC lesion [32].

A treatmenet algorithm for ulnar-sided TFCC injuries has been proposed, in which treatment depend on both the completeness of the lesion (dc-TFCC or/and pc-TFCC) as well as the condition of the TCFF (repairable or non-repairable) [8]. Complete repairable combined dc- and pc-TFCC (class 2) can be surgically treated by open or arthroscopic foveal TFCC reinsertion. Contrary, delayed diagnosis of complete TFCC tears may result in a chronic (> 6 months) [7] non-repairable TFCC tear (class 4) with degenerative retracted edges and poor healing potential [6, 7, 23]. These injuries require surgical TFCC reconstruction with a tendon graft [2, 7]. It is unknown if these surgical methods perform equivalently in terms of regaining primary DRUJ stability.

Investigations of the stabilizing effect of different surgical methods should preferably be performed in cadaver studies prior to clinical introduction. A non-invasive method for automated radiostereometric analysis (AutoRSA) was recently shown to provide precise quantification of DRUJ translation during the Piano-key test in cadavers [30].

The aim of this experimental study in human cadaver arms was to compare the effect of; open surgery with foveal reinsertion of the TFCC, or ligament reconstruction of the TFCC with palmaris longus graft ad modum Adams on the primary stability of the DRUJ.

## Methods

### Study design and specimens

We conducted a parallel group randomized controlled trial on human donorarms. The primary outcome in this experimental cadaver study was translation in the DRUJ during the Piano-key test. Ten freshly frozen human donorarms including hand, forearm, elbow and part of the humerus were used (Department of Biomedicine, Aarhus University). They were thawed for 48 h at 5 °C before use in the study.

The specimens (eight men, mean age 78 years (range 63–90)) were evaluated at baseline and met the inclusion criteria: no signs of previous fracture or malunion as evaluated by fluoroscopy of the wrist, forearm and elbow. The Central Denmark Region Committees on Health Research Ethics approved the study (Casenr. 1–10-72–6-16 issued on February 24th, 2016).

### Experimental setup

A radiolucent motorized fixture was used [30]. It allowed for a 7 kg load to be applied on the ulnar head by use of a fixture lever to imitate the clinical Piano-key test examination in a standardized setting [11]. An quivalent force was described not to give “obvious disruption of the soft tissues” in an experimental study [29]. In the test set-up the humerus was fixed in a 90 degrees vertical position, the forearm was pronated, and the hand was fixed to a horizontal plate, in zero degrees wrist extension and wrist deviation [30].

### Test protocol

Ligament lesion of the TFCC was performed using fluoroscopic visualization. The dorsal DRUJ capsule was opened transversely proximal to the TFCC, the dc-TFCC was released from the insertion on the ulnar styloid and the pc-TFCC was cut from the insertion in the ulnar fovea. Additional soft tissue and the remaining TFCC stabilizers of the DRUJ, including the interosseous membrane, were preserved.

Clinical examination of all specimens before and after intervention (Piano-key test and Ballottement test) was performed by two hand surgeons and consensus was obtained. DRUJ instability was evaluated as translation with the Ballottement test and categorized as proposed by Atzei et al.: less than 5 mm, between 5–10 mm (mild instability) or above 10 mm (severe instability) [9].

Wrist artrhroscopy was performed to confirm dc- and pc-TFCC lesion in terms of a positive trampoline test [15] and a positive Hook test [6].

For evaluation of DRUJ translation, the specimens were positioned in the custom-made fixture and recorded with synchronized static stereoradiographs before and after applying the Piano-Key test. The test was done twice on the specimens: first, after inflicted dc- and pc-TFCC lesion, and second, after surgical intervention.

### Intervention

The specimens were randomly assigned to one of two treatment groups. The open foveal TFCC reinsertion group was treated by open surgery: The skin was incized dorsal over the DRUJ and the DRUJ capsule was exposed through the 5^th^ extensor compartment, leaving the most distal part of the extensor retinaculum intact. A L-shaped capsular opening was performed by extending the opening to the radial side of the extensor carpi ulnaris tendon sheat on the proximal aspect of the dorsal radioulnar ligment, preserving the radial insertion. Any DRUJ synovitis was removed, the fovea was identifyed and controlled by fluroscopy before drilling and inserting a 2–0 Mitec Mini QUICKANCHOR® (DePuy Mitek, Raynham, MA, USA). The pc-TFCC was reinserted by a matress suture through the TFCC from proximal to distal and the Mitec suture was tied with 5 knots while the assistant compressed the DRUJ in neutral forearm rotation. Finaly, the dorsal capsule and the skin was closed with 3–0 vicryl sutures.

The Adams TFCC reconstruction group was reconstructed with a palmaris longus graft as described by Adams [1]. The DRUJ capsule was exposed through the 5^th^ extensor compartment and an L-shaped capsular flap. Placement of the radius tunnel was guided by fluroscopy: a k-wire was placed for over-drilling of a 4 mm tunnel proximal to the lunate fossa and radial to the articular surface of the sigmoid notch. Likewise, a k-wire guided oblique ulnar tunnel was drilled from the lateral ulnar neck and emerging in the ulnar fovea. The palmaris graft was harvested and passed from the dorsal to the volar aspect of the wrist through a volar incision extending 3 cm promimal from the proximal wrist crease. The volar aspect of the radial tunnel was exposed and the graft was retracted with a straight tendon grasper. The volar limb of the graft was passed through the DRUJ capsule proximal to the TFCC remnants and both tendon limbs were passed through the ulnar tunnel. Finaly, the volar tendon limb was passed volarly around the ulnar neck, close to the bone, and tied dorsally with the first half of a surgeons knot while the assistant compressed the DRUJ in neutral forearm rotation. The tendon knot was secured with three 3–0 fiberwire mattress sutures. In addition a second tendon knot was tied and secured with further three mattress sutures. Finaly, the dorsal capsule and the skin was closed with 3–0 vicryl sutures.

### Randomization

The specimens were numbered and subsequently randomized by sequential drawing of ten sealed opaque envelopes, prepared with an equal 1:1 ratio distribution of intervention labels, that randomly assigned the specimens to two intervention groups: open surgery with foveal TFCC reinsertion [15]; or Adams TFCC reconstruction, with palmaris longus graft [1].

### Static radiostereometry setup

**Fig. 1:**
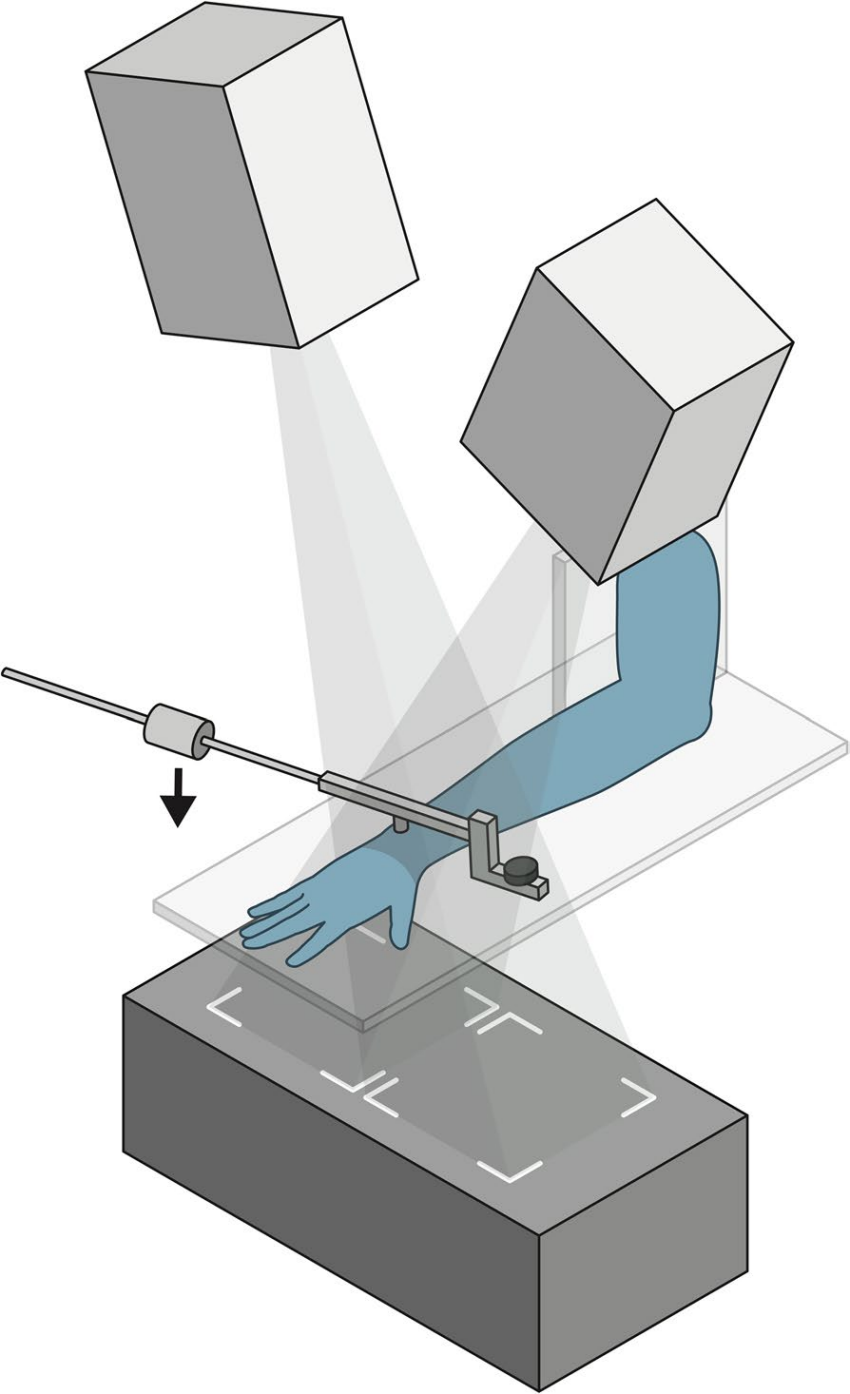
Digital radiostereometric system setup*.* The x-ray tubes are positioned with a 20°-20° tilt on the vertical plane. Static wrist examinations were recorded with two digital image detectors (Canon CXDI-50RF) beneath a horizontal positioned uniplanar carbon calibration box. The Piano-key test is indicated by the arrow and appyed by a lever (7 kg)

A digital radiostereometric system (AdoraRSA, NRT X-Ray, Hasselager, Denmark) was used to record static examinations of the specimens. Images were obtained with two digital image detectors (Canon CXDI-50RF) slotted beneath the uniplanar carbon calibration box (Carbon box 19, Medis Specials, Leiden, The Netherlands) and exposed with two x-ray tubes (20°-20° tube position on the vertical plane) (Fig. 1). Exposure settings for static stereoradiographs were 60 kV, 2.5 mAs, 2208 × 2688 pixels resolution (0.16 × 0.16 mm/pixel). The Source Skin Distance (SSD) was 100 cm and the Source to Images Distance (SID) was 150 cm.

### Analysis of radiographs

Analysis of the static stereoradiographs depend on bone models and kinematic axis. The bone models were generated form computer tomography (CT) scans (Philips Brilliance 64, 120 kV, 100 mAs) of the intact human donor forearms. CT images were reconstructed (0.9 mm slice thickness, 0.45 mm slice increment and 0.27 × 0.27 mm in-plane pixel size) and The Insight Segmentation and The Visualization Toolkit softwares (Kitware, New York, USA) were used for image processing of subject specific bone models (radius and ulna). First, an automated graph-cut method was used for bone segmentation. Second, bone volume models with greyscale information were extracted. Surface bone models were created and finaly simplified to consist of 10.000 triangles [14].

**Fig. 2:**
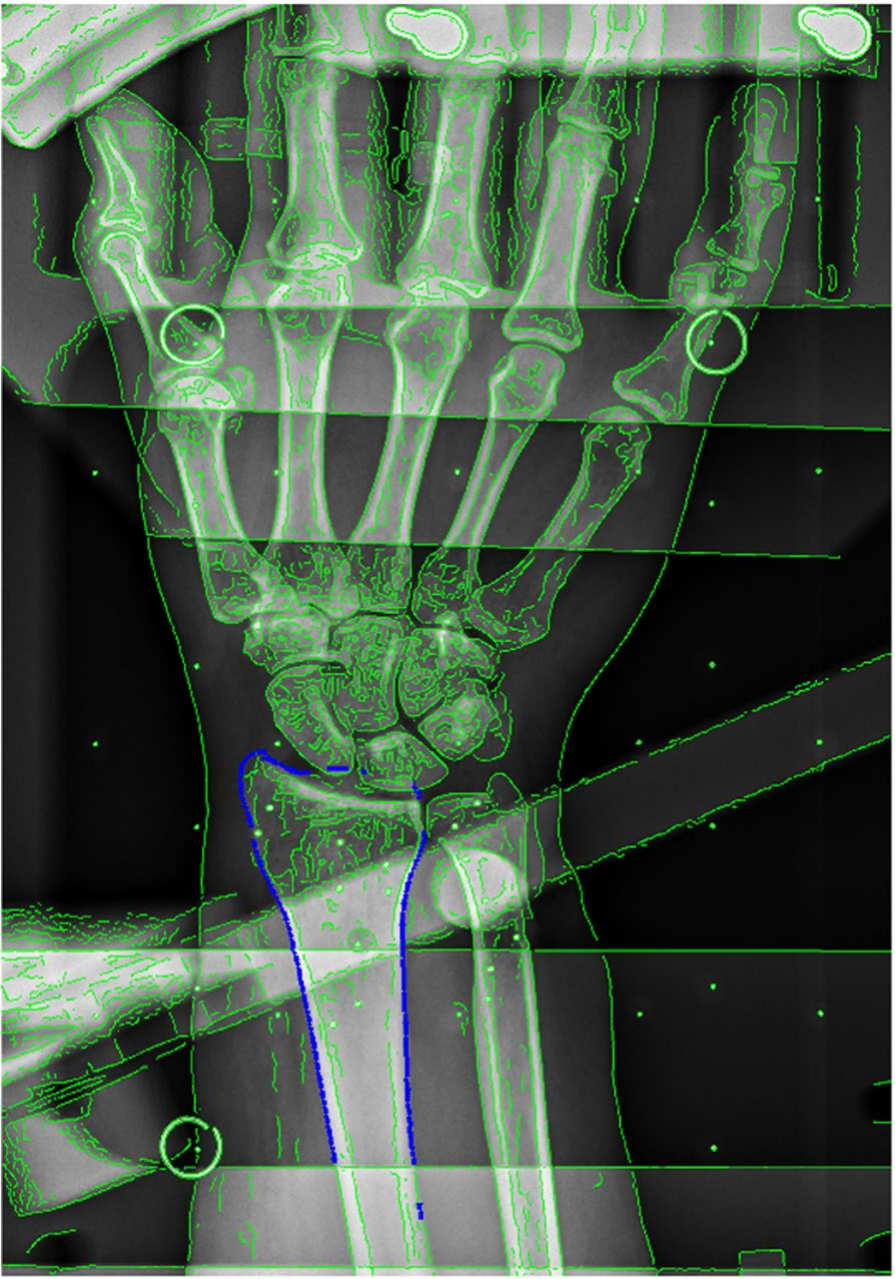
Model-based radiostereometric analysis (MBRSA)*.* MBRSA software automatically detected the ulna and radius bone edges (green) and relevant edges (blue) was manually selected on the stereoradiographs

Analysis of the stereoradiographs defined the three-dimensional position and orientation of the ulna and radius bone. Model-based radiostereometric analysis software (MBRSA 4.11, RSAcore, Leiden) was used for image calibration. Further, the Model-based RSA software automatically detected the bone edges of the ulna and radius on the stereoradiographs and the relevant edges were selected manually [18] (Fig. 2). The CT based surface bone models were imported in the program and the best pose of the bones was automatically estimated by minimizing the error of the surface bone model projections versus the manually detected bone edges on the stereoradiographs. The final pose was used as an initial bone position in the subsequent analysis of the stereoradiograph with non-commercial AutoRSA software.

**Fig. 3:**
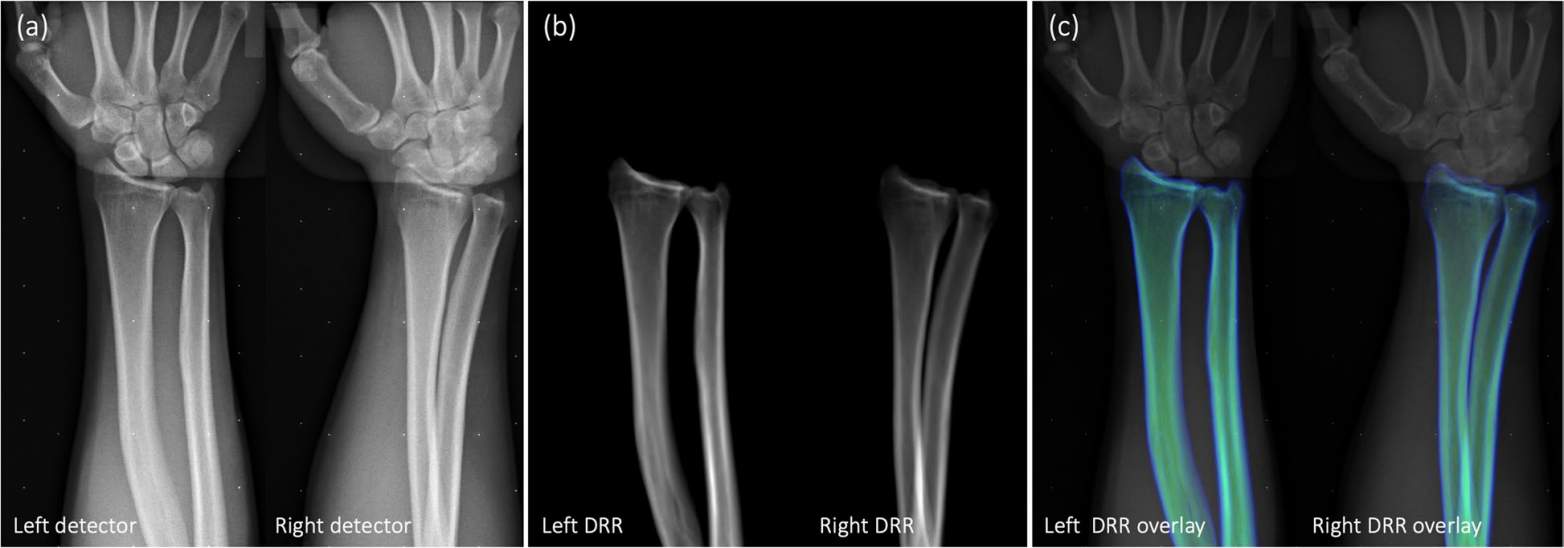
AutoRSA analysis of radiostereometric images. Comparison of (**a**) radiostereometric images and (**b**) CT based digital reconstructed radiographs (DRR), was performed with a mathematical algorithm in the AutoRSA software until no further improvements could be made. The optimal overlay (**c**) was calculated by the AutoRSA software

The CT based volume bone models were used to simulate digital reconstructed radiographs (DRR), and the AutoRSA software calculated the optimal pose of the models by repeated comparison between the simulated DRR and the stereoradiographic images until no further improvements could be made (Fig. 3). The bone registration area was focused on the stereoradiograph with an automatically produced mask projected from the CT bone volume model.

We have previously examined the precision of the AutoRSA software, as compared to marker-based radiostereometric analysis (reference standard), for dynamic examinations of the radius and ulna. The precision of AutoRSA (95% Limits of agreement) was below 0.12 mm for translation of the radius, below 0.18 mm for translation of the ulna, and less than 0.98 degrees in rotations for both the radius and ulna.

### Coordinate system and kinematic axis

The position of the radius and ulna in the calibration box coordinate system was transformed to a standardized anatomical coordinate system for each bone. Three orthogonal axes (x,y,z), were each defined from three anatomical landmarks [20] on the 3D CT of each bone surface model. The radius landmarks were; the proximal rotation center of the radial head (C_prox_), the radial styloid tip, and the distal radioulnar joint surface center. On the ulna the landmarks were; the ulnar head center (C_dist_), the distal ulnar styloid tip, and greater sigmoid notch center. The best fitted sphere of 3 points picked on the radial and ulnar head surfaces was used to compute the center points.

**Fig. 4:**
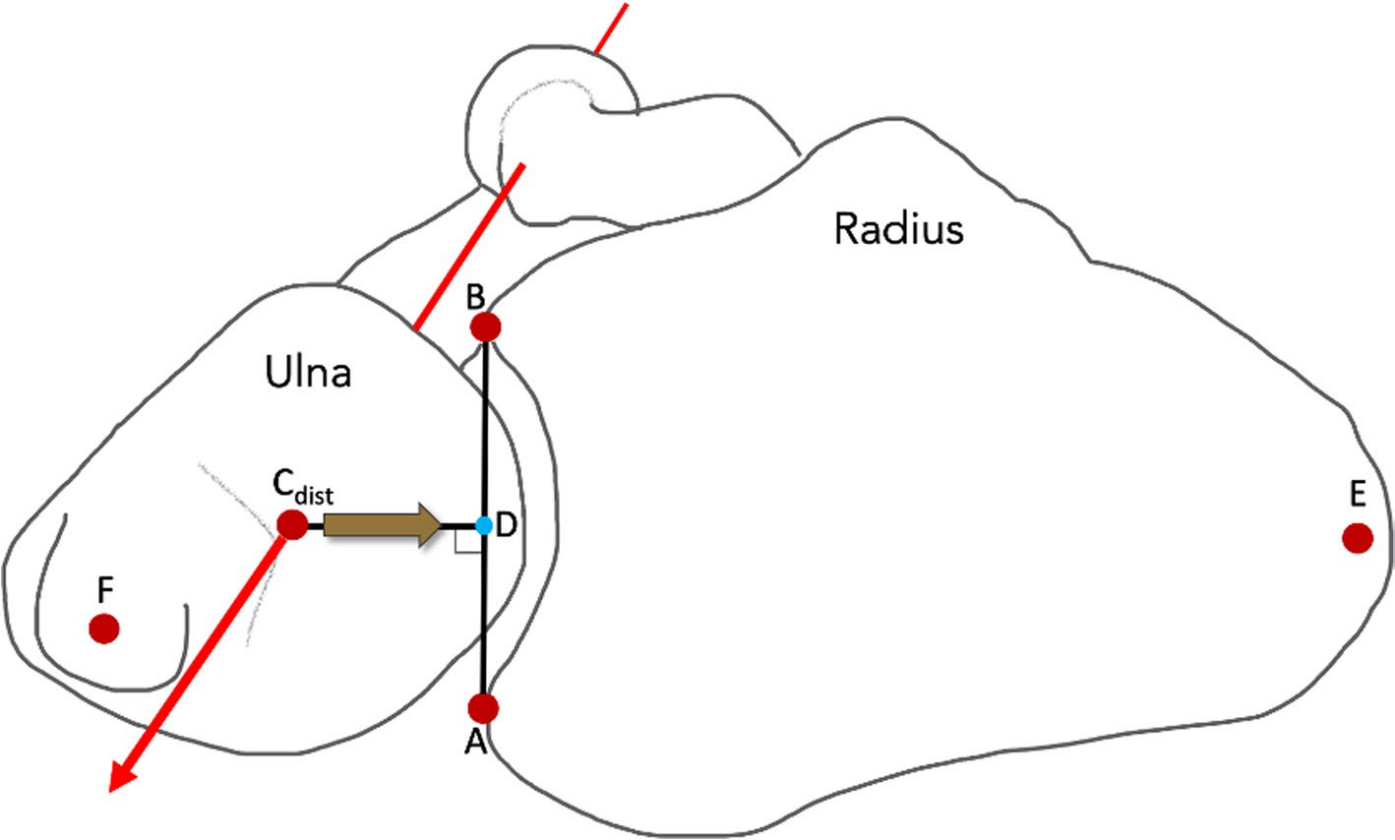
Kinematic axis and anatomical landmarks*.* The distal radioulnar (DRUJ) position (D) was defined as the orthogonal projection (yellow arrow) from the radioulnar axis (red line) perpendicular to the radius sigmoid notch line (AB) connecting the anterior (A) and posterior (B) rim points. The DRUJ translation was calculated as the change of DRUJ position (D) on the sigmoid notch line (AB) in millimeters. The DRUJ postion ratio was calculated as AD/AB. Forearm rotation was calculated as; the angle between the line from the radial styloid tip (E) to the midpoint on the sigmoid notch line (AB), and the line from the ulnar head center (C_dist_)  to the distal ulnar styloid tip (F)

**Fig. 5:**
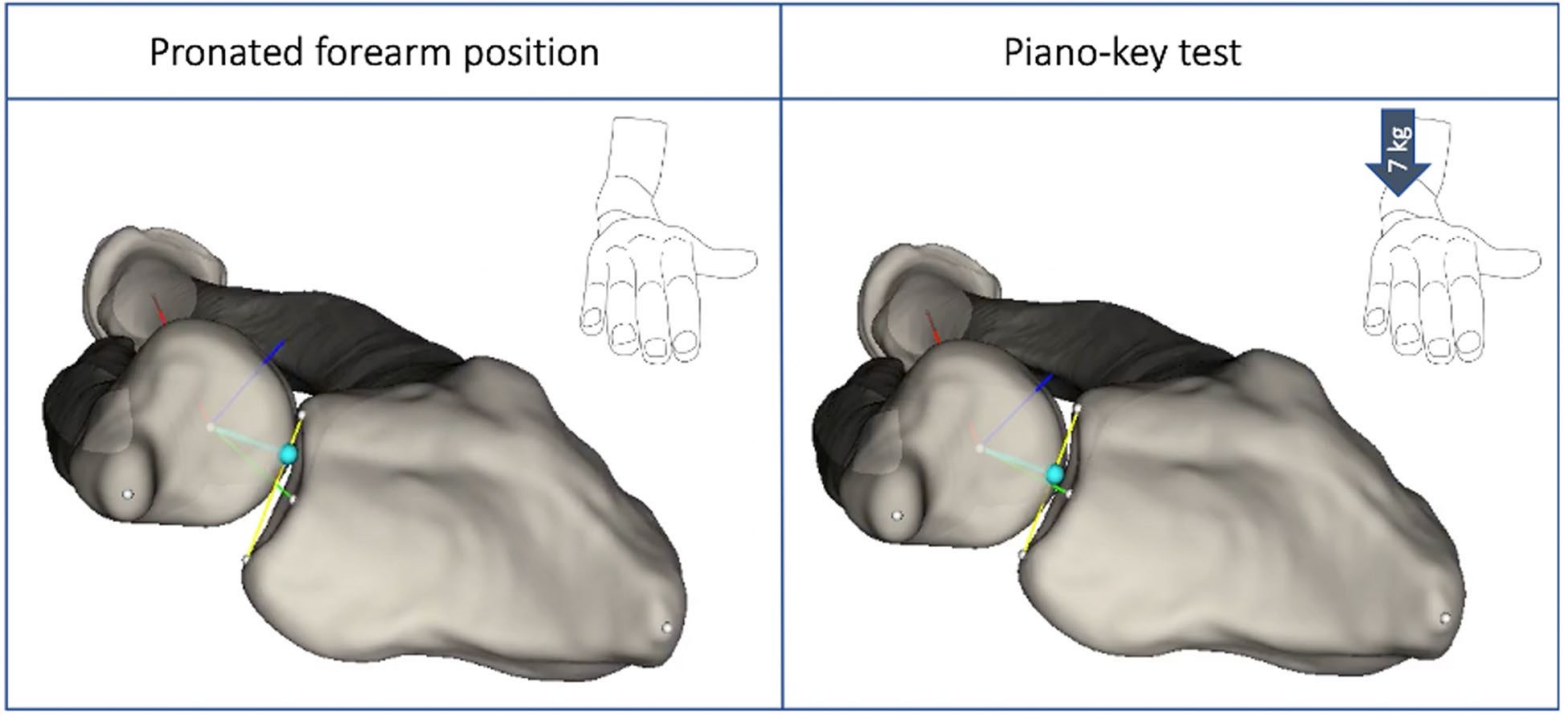
Bone model position after AutoRSA analysis of a cadaver arm before and after the Piano-key test

A single radioulnar joint axis (RUJ axis) extending from the radial head centre to the ulnar head centre as described by Hagert et al. was used to calculated kinematics [20]. Further, the radius sigmoid notch line, a connecting line from the midpoint of the volar to dorsal rim of the radius sigmoid notch, was definedThe orthogonal projection of the RUL axis on the radius sigmoid notch line determined the DRUJ position. The DRUJ position ratio was calculated as the relation of the DRUJ position and the individual sigmoid notch length, to take the difference of individual bone-sizes into account. The DRUJ translation was the change of DRUJ position in millimeters (Figs. 4 and 5).

Forearm rotation was calculated as; the angle between the line from the radial styloid tip to the midpoint on the sigmoid notch line, and the line from the ulnar head center and to the distal ulnar styloid tip (Fig. 4).

### Sample size

The sample size calculation was based on a study by Pickering et al. who used an externally mounted rig to measure DRUJ translation on pronated forearms in normal and clinically unstable populations [5]. The DRUJ translation on the pronated forearm was 4.2 mm (SD 0.5) in healthy controls compared to 7.0 mm (SD 0.5) in the clinically unstable patient group. With a power of 0.90 and alfa of 0.05 a sample size of three patients per group for a two-sample comparison of means was estimated. A sample size of five patients per group was selected to allow for incomplete data collection/imaging errors.

### Statistical analysis

Categorical data was reported as numbers and were compared between groups using the chi-squared test. Normality of continous data was evaluated by instpection of frequenzy and probability plots (quantile–quantile plots). The student’s paired t-test was used to compare forearm rotation, DRUJ position and DRUJ translation before and after intervention within groups. Comparison between the independent groups were performed with the non-paired t-test. The level of significance was set at *p* < 0.05 and data was reported as means and 95% confidence intervals (95% CI).

## Results

### Preoperative group comparison

The two groups had comparable preoperative characteristics including age, sex, right/left hand, clinical instability evaluation with the Ballottement test and arthroscopic evaluation (Table 1).

**Table 1:** Specimen characteristica

	**Foveal reinsertion**	**Adams reconstruction**	***p***
**Number**	5	5	
**Age in years (**mean, range**)**	77 (72–90)	79 (63–90)	0.98
**Sex** (men/women)	5/0	3/2	0.11
**Side** (right/left)	4/1	1/4	0.06
**Ballottement test **^**a**^			
*** Neutral position***	0/2/3	0/4/1	0.29
*** Supination***	3/2/0	4/1/0	0.49
*** Pronation***	0/5/0	0/5/0	1.0
**Trampoline test (-/ +)**	0/5	0/5	1.0
**Hook test (-/ +)**	0/5	0/5	1.0

Summarized characteristica and pre-operative clinical- and arthroscopic findings of cadaver wrists with combined dc- and pc-TFCC lesion

^a^Numbers evaluated with less than 5 mm, between 5–10 mm (mild instability) or above 10 mm DRUJ translation (severe)

### Clinical examination

After combined TFCC lesion, a consensus evaluation between two hand surgeons categorized all 10 cadaver arms with > 5 mm translation in the DRUJ during the Ballottement test on neutral forearm rotation. 

Both the foveal TFCC reinsertion and the Adams TFCC reconstruction stabilized the DRUJ, as the Ballottement test on neutral forearm rotation, was categorized to translate less than 5 mm in all 10 cadaver arms, after surgical treatment (Table 1).

### Atrhroscopic evaluation

The preoperative arthroscopic evaluation revealed a positive Trampoline test and Hook test in all ten cadaver arms after ligament lesion including the dc- and pc-TFCC (Table 1).

### Preoperative radiostereometric evaluation

The DRUJ position ratio in pronated forearms (*n* = 10) with inflicted dc- and pc-TFCC lesion was mean 0.68 (95% CI 0.61; 0.75). The Piano-key test induced a dorso-volar DRUJ translation of mean 18% (95% CI 12; 25) of the sigmoid notch length, cooresponding to 2.45 mm (95% CI 1.68; 3.22).

**Fig. 6:**
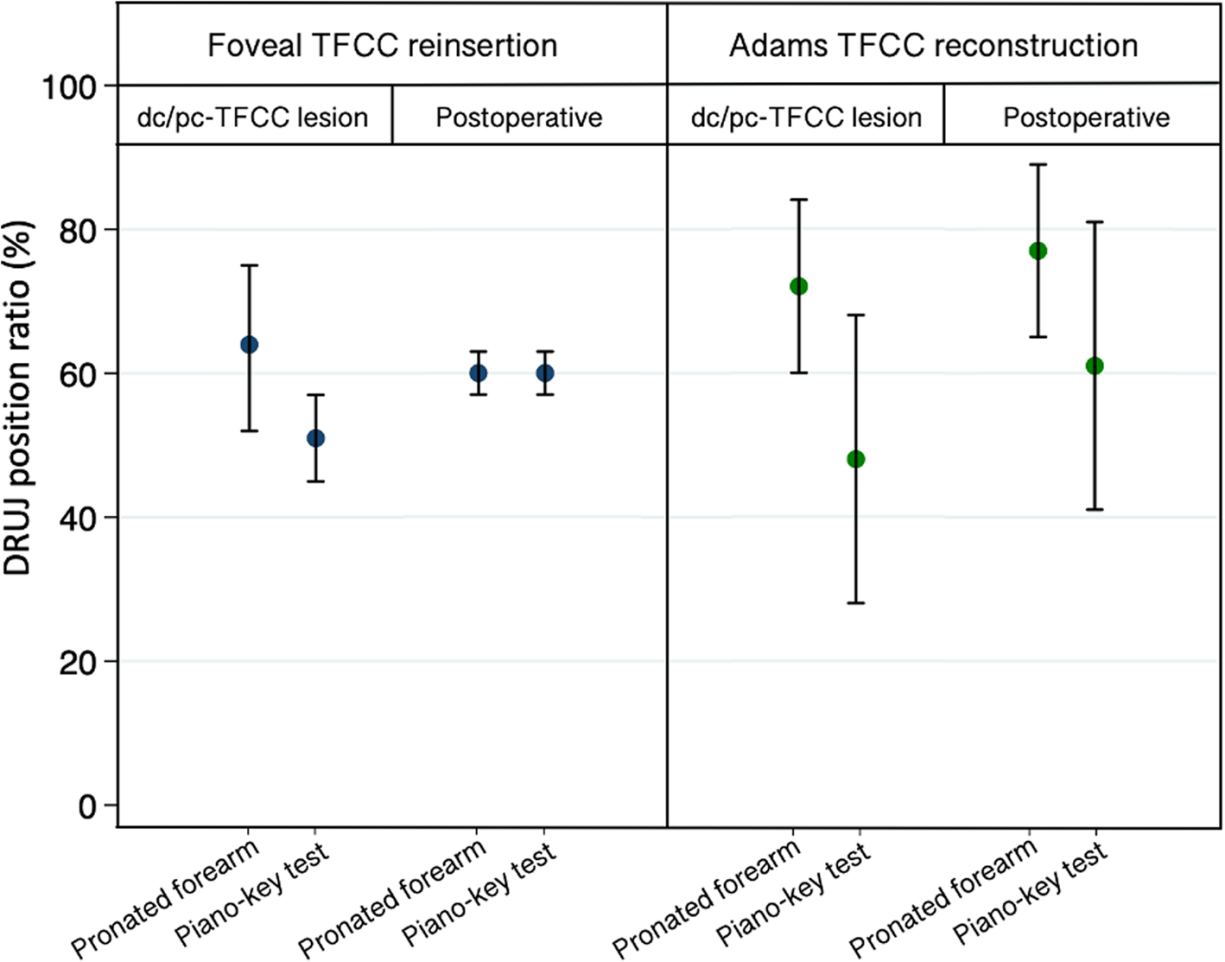
DRUJ position ratio (%) in the foveal TFCC reinsertion group (*n* = 5) and the Adams reconstruction group (*n *= 5), on pronated forearm and during the Piano-key test, with combined distal- and proximal component TFCC lesion and after surgical treatment. (DRUJ: Distal radioulnar joint, dc: distal component, pc: proximal component, TFCC: triangular fibrocartilage complex; dc-TFCC: distal component TFCC; pc: proximal component TFCC)

A comparison of the foveal TFCC reinsertion and Adams TFCC reconstruction groups with inflicted dc- and pc-TFCC showed no difference in DRUJ position ratio before apying the Piano-key test (*p* = 0.21). In both groups the Piano-key test induced a statistically significant volarly directed translation of the ulnar head in the sigmoid notch (*p* < 0.01) (Fig. 5). The resulting DRUJ position was mean 0.51 (95% CI 0.45;0.57) and mean 0.48 (05% CI 0.28;0.68), respectively (*p* = 0.72) (Table 2, Fig. 6).

**Table 2:** Specimens distal radioulnar joint pronation and position ratio

*Group*	**With dc/pc-TFCC lesion**			**After surgical treatment**		
	*Foveal TFCC reinsertion*	*Adams TFCC reconstruction*	*p*	*Foveal TFCC reinsertion*	*Adams TFCC reconstruction*	*p*
Number	5	5		5	5	
**Pronated forearm**						
Degrees pronation (°)	81 (68–93)	82 (72–91)	0.87	58 (44–73)	68 (49–88)	0.31
DRUJ position ratio	0.63 (0.52–0.75)	0.72 (0.60–0.84)	0.21	0.60 (0.57–0.63)	0.77 (0.65–0.89)	0.005
**Piano- key test**						
Degrees pronation (°)	68 (61–76)	59 (53–65)	0.02	60 (44–76)	60 (45–69)	0.68
DRUJ position ratio	0.51 (0.45–0.57)	0.48 (0.28–0.68)	0.72	0.60 (0.57–0.63)	0.61 (0.41–0.81)	0.87

Degrees of forearm pronation and DRUJ position ratio before and after the Piano-key test in cadaverarms with combined dc- and pc-TFCC lesion and after surgical repair with foveal TFCC reinsertion or Adams TFCC reconstruction. Data are presented as means and (95% CI)

*DRUJ* Distal radioulnar joint, *dc* distal component, *pc* proximal component, *TFCC* triangular fibrocartilage complex

**Fig. 7:**
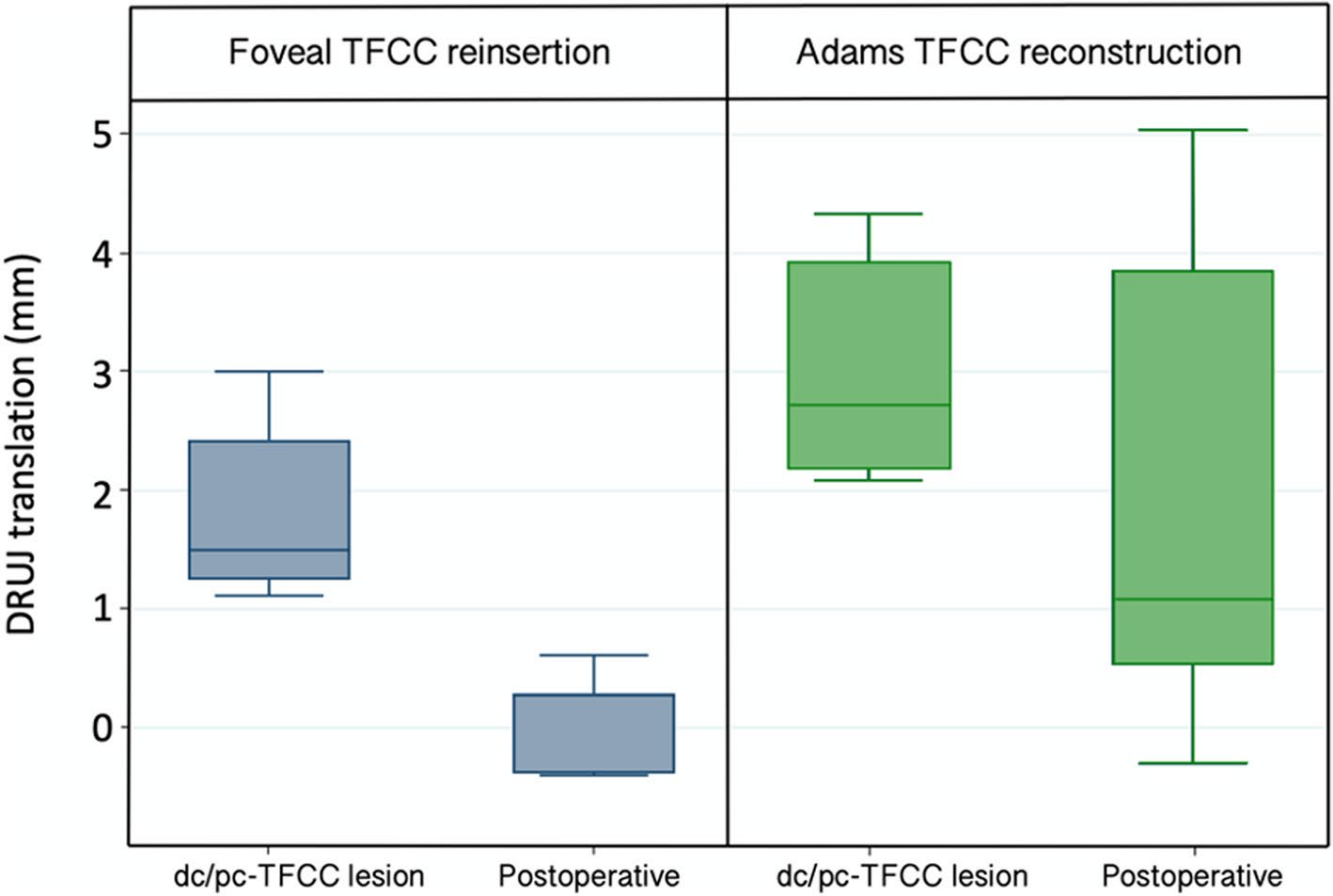
Box plot of DRUJ translation (mm) induced by the Piano-key test in the foveal TFCC reinsertion group (*n* = 5) and the Adams reconstruction group (*n* = 5), with combined dc/pc-TFCC lesion and after surgical treatment. (DRUJ: Distal radioulnar joint, dc: distal component, pc: proximal component, TFCC: triangular fibrocartilage complex)

The preoperative DRUJ translation induced by the Piano-key test was mean 1.86 mm (95% CI 0.84; 2.89) in the foveal TFCC reinsertion group and mean 3.05 mm (95% CI 1.78; 4.32) in the Adams TFCC reconstruction group (*p* = 0.08) (Fig. 7).

With lesion of the dc- and pc-TFCC, the maximum passive forearm pronation in the test fixture was mean 81 degrees (95% CI 68; 93) in the FR group and mean 82 degrees (95% CI 72; 91) in the Adams TFCC reconstruction group (*p* = 0.87) (Table 2).

### Postoperative radiostereometric evaluation

Surgical treatment did not shift the DRUJ position ratio of the pronated arm significantly in either group (*p* > 0.30). The Piano-key test shifted the ulnar head to a similar DRUJ position ratio of mean 0.60 (95% CI 0.57; 0.63) in the foveal TFCC reinsertion group and to mean 0.61 (95% CI 0.41; 0.81) in the Adams TFCC reconstruction group (*p* = 0.87) (Table 2, Fig. 6).

Surgical treatment reduced the DRUJ translation by mean 1.78 mm (95% CI 0.82; 2.74) in the foveal TFCC reinsertion group (*p* = 0.007), and by mean 1.01 mm (95% CI -1.58; 3.60) in the Adams TFCC reconstruction group (*p* = 0.17) (Fig. 7). The stabilizing effect of the two surgical methods was similar (*p* = 0.31), but with greater variation in the Adams TFCC reconstruction group.

The final DRUJ translation induced by the Piano-key test after surgery, was mean 0.08 mm (95% CI -0.48; 0.64) in the foveal TFCC reinsertion group and mean 2.04 mm (95% CI -0.81; 4.89) in the Adams TFCC reconstruction group (*p* = 0.10) (Fig. 7).

Surgery reduced the passive pronation with mean 23 degrees (95% CI -3; 46) in the foveal TFCC reinsertion group (*p* = 0.07) and with mean 14 degrees (95% CI -5; 32) in the Adams TFCC reconstruction group (*p* = 0.12) (Table 2). The decrease in pronation was similar in the two groups (*p* = 0.46).

## Discussion

In the present study, we found a mean DRUJ translation after Adams TFCC reconstruction of mean 2.03 mm (95% CI -0.81; 4.89).

Objective measuring tools useful for clinical assesment of DRUJ stability in surgically treated patients are few, and to our knowledge, Hess et al. is the only other research group who have developed, validated and used an objective measuring tool, for assessment of DRUJ stability in surgically treated patients [16]. They treated 11 patients with open TFCC reconstruction similar to the Adams [1] method, but with a modification of the graft fixation, and used ultrasonography to evaluate the DRUJ translation of the operated wrist in comparison with the contralateral healthy wrist. After TFCC reconstruction the uni-directional sonography measured DRUJ translation was mean 3.5 mm (range 1.1–6.2) [16]. Yet, a marked variation in stabilization effect was seen as the DRUJ translation was decreased in three patients, another three had DRUJ translation comparable to the contralateral healthy wrist, and the remaining five patiens were still more lax than on the contralateral side. This is in accordance with the present study as we observed high variability of the stabilizing effect of the Adams ligament reconstruction and no significant improvement of the DRUJ translation.

Contrary, open foveal TFCC reinsertion stabilized the DRUJ significantly and homogenuously with a mean DRUJ translation of 0.08 mm (95% CI -0.48; 0.64). However, the method tended to reduce the DRUJ translation to nearly zero. In a previous study on uninjured cadaver wrists with normal arthroscopic Hook test and trampoline test, examined with a similar radiostereometry setup, we found a DRUJ translation of mean 1.36 mm (95% CI 0.17;2.55) [30]. It is unknow if overtightening of the radioulnar ligaments during TFCC surgery will obstruct the rehabilitation of supination and pronation motions or result in pain. Hess et al. reported poor patient reported outcomes (PRWE) and persisting wrist pain in one patient with decreased DRUJ translation compared to the contralateral side, but the forearm rotation was acceptable [16].

### Clinical evaluation of DRUJ stability

In this study, clinical examination of DRUJ instability was assesed with the ballottement test.

We did not have a contralateral arm to compare to, as recommended by Nakamura et al. [23]. Therefore, we categorized the DRUJ instability grade as proposed by Atzei et al. [9]. The postoperative DRUJ translation was graded to be less than 5 mm in all cadaver arms with no difference between the foveal TFCC reinsertion and Adams TFCC reconstruction groups. Thus, the difference of surgical methods on the effect of DRUJ stability was only detectable with radiostereometry.

In patients, abnormal translation with a ‘soft’ resistance can be felt in the clinically unstable DRUJ [7]. However, muscular stabilizers of the DRUJ can lead to a false negative examination in DRUJ unstable patients [6]. Clinical wrist examination has previously been described as subjective, highly observer dependent, and of limited diagnostic value to detect TFCC lesions [27]. This may contribute to the problem of delayed diagnosis of DRUJ instability after wrist fractures and/or sprains [4], as well as to challenge a reliable objective evaluation of DRUJ stability in the postoperative phase. Despite this fact, surgeons most frequently use clinical examination for postoperative evaluation of DRUJ stability in clinical studies [2, 21, 23], whereas precise and validated objective examination tools are rarely used.

### Other methods for evaluation of DRUJ stability

In-vivo methods for diagnosing DRUJ instability are available. Computer tomography (CT) of static forearm supination and pronation have been used to detect DRUJ instability in terms of subluxation, but the reliability of these static methods vary and do not asses the DRUJ translation [25]. Pickering et al. developd and used an externally mounted rig for examination of 50 patients with TFCC lesions, and found a bi-directional translation of 7.0 mm (SD 0.5) in pronated forearms [26]. Hess et al. used ultrasonography for preoperative examination of in 17 patients with TFCC lesions, and measured a uni-directional DRUJ translation of mean 5.1 mm (range 2.4–7.1) [17].

With devices only applicable for ex-vivo use the bi-directional DRUJ translation in pronated forarms was repored to range from 2.9–12.4 mm [19, 24, 28].

In the present study the uni-directional DRUJ translation was 2.45 mm (95% CI 1.68; 3.22) in cadaverarms with combined distal component and proximal component TFCC lesion. This is less than previous reports, which may be explained by differences in bi/uni-directional measures, soft tissue movement being included in the rig measures, and the degree of pronation during examination.

A clinical applicable method including measures of bone and joint kinematics only, is preferable and increase realiability in small joints.

### DRUJ position ratio

The native DRUJ was previously described to be stabilized in pronation by the bony sigmoid notch concavity [3] and moreover, by the proximal component of the TCFF which insert in the fovea [12, 22, 29]. In a previous radiostereometric study on intact cadaverarms, the DRUJ position ratio was 0.61 (95% CI 55;67) when applying the Piano-key test, which is comparable to the final DRUJ position ration obtained after surgery in both the foveal TFCC reinsertion and Adams TFCC reconstruction groups in the present study [30].

### Limitations

This experimental study was performed on an aged cadaver population and has natural limitations. Post-mortem ligament laxity and tensile strength as well as the type of TFCC lesion that can be applied ex-vivo do probably not completely resemble the conditions of in-vivo traumatic TFCC lesions and the resulting pre-operative group instability varied despite randomization.

Efforts were made to standardize the test set-up by performing fluoroscopy assisted ligament lesion, and all specimens had similar clinical assessment and arthroscopic verification of a positive Hook test was performed before RSA examination (Table 1). Despite this, the sample size may not have been sufficiently large to ensure high preoperative similarity or sufficiently large to detect significant differences in stability gained by the surgical procedure (type 2 error).

We performed pre-study fluoroscopy and CT scans of the used specimens and excluded any with visible fracture deformity, which could influence the DRUJ kinematics. In addition, arthroscopy was used to confirm and classify TFCC lesions like in the clinical situation. The original method of TFCC reconstruction, described by Adams et al. was used [1]. The final palmaris graft closure depend on knots and suturing of the graft. The tecnique has been modified by other authors to replace tendon knots with an intereference screw to secure the tendon graft in the ulna bone, which may produce more reliable DRUJ stability [16, 31].

This study is experimental and can only account for the stability of the surgical techniques directly after surgery. In patients, the effects of adhesions, scar tissue generation and developed laxity during rehabilitation, may affect DRUJ stability after longer-term clinical follow-up.

## Conclusions

This study demonstrates the feasibility of radiostereometric imaging and AutoRSA analysis in an experimental setup, a non-invasive CT bone model-based method, for precise quantification of DRUJ translation before and after surgical treatment.

Dynamic radiostereometry and AutoRSA analysis is an innovative method that has been proven feasible for studies of kinematics of other joints [10, 13]. In a clinical perspective, a valid imaging and analysis method for examination of DRUJ translation in patients is demanded. The AutoRSA method is likely applicable in patients during dynamic loaded tests for evaluation of DRUJ translation in a diagnostic assessment and after surgical treatments. Investigations of feasibility and validity in patients and establishment of normal values for DRUJ stability are warranted.

In conclusion, the open foveal TFCC reinsertion to the ulnar fovea provided a significant decrease in DRUJ translation with foveal TFCC reinsertion, whereas the stabilizing effect of the Adams TFCC reconstruction had greater variation and demonstrated no significant improvement of the DRUJ translation.

This supports the current clinical recommendation of TFCC reinsertion in patients suffering from symptomatic DRUJ instability due to acute fovea TFCC lesions and emphazice the importance of timely diagnosis and treatment. On the contrary, this also reinforce the recommendation that TFCC reconstruction should be spared for treatment of chronic lesions, where the remnant of the TFCC is absent or too weak to be repaired.

However, the clinical relevance of the observed difference has to be studied in a clinical setup with focus on the stabilizing effect on patient reported outcome.

## Data Availability

The datasets used and/or analysed during the current study are available from the corresponding author on reasonable request.

## Abbreviations

DRUJ: Distal radioulnar joint
TFCC: Triangular Fibrocartilage Complex
pc: Proximal component
dc: Distal component
CT: Computer tomography
AutoRSA: Automated radiostereometric analysis
SSD: Source Skin Distance
SID: Source to Images Distance
DRR: Digital reconstructed radiographs
C_prox_: Proximal rotation center point of the radial head
C_dist_: Ulnar head center point
RUJ: Radioulnar joint
PRWE: Patient-Rated Wrist Evaluation
